# Impaction of Molar Tooth in Left Bronchus, Etc.

**Published:** 1871-06

**Authors:** 


### ARTICLE XII.

### *Impaction of Molar Tooth in Left Bronchus, etc.*

Dr. Weir presented a specimen, showing the impaction of a greater portion of the molar tooth in the first division of the left bronchus. The patient from whom it was removed was an inmate of St. Luke's Hospital. Some months ago, he sustained a compound fracture of the lower jaw, the result of a blow from an axe. The ordinary treatment was employed, but the union was very imperfect, owing to the impaction of some dead bone. It being thought necessary to remove the dead bone referred to, the patient, a stout pale Irishman aged forty, was etherized Jan. 3. He took the ether very badly. Enlarging the fistula which led to the dead bone, it was discovered that a sequestrum was attached to a molar tooth. The tooth was seized, and, in the attempt to withdraw it, the patient was seized with a vomiting-fit, and in the struggle the tooth being dislodged, disappeared. The material vomited was in the confusion thrown away by the attendant. Not being satisfied as to the whereabouts of the tooth, the house-surgeon was directed to watch the patient, and to have the fæces thoroughly examined. This was done with no result. There was no marked coughing, or evidence of irritation about the chest when the tooth disappeared. For some days after, the patient did not do very well, but this circumstance was attributed to the amount of blood lost. In the course of two or three days he had a slight attack of bronchitis, but on examination nothing was found. Under the usual treatment the symptoms abated. Nothing of special note occurred in the case until the tenth day after the operation, when the house-surgeon reported that with a persistent bronchial irritation, the patient had very feeble respiration of the left side. At times the inspiration over the left lung would appear to be suddenly checked, although expiration seemed at all times easy. A consultation having been called on another case, the gentleman present saw this one also. On examination no satisfactory results were obtained, save that the existence of pneumonia was recognized, the only thing peculiar about the case being a heaving effort at respiration to which attention was called by Dr. Sands. It was deemed advisable to perform tracheotomy as an explorative operation, and then attempt to dislodge the tooth if it could be reached farther down, thus repeating an operation of a similar nature so successfully performed by Dr. Buck. The patient however refused to have the attempt made, and died unrelieved.

At the autopsy the left lung was found the seat of red hepatization, and in the first division of the bronchial tube was found a decayed molar, the upper portion of which was hooked into the mucous membrane in such a manner as to preclude the possibility of its exaction from above in case the operation has been performed. About an inch below the point where the tooth was found, and adjoining the pleura, was an abscess filled with broken-down tissue, the whole being about the size of an English walnut; another, also of the same size, was found deeper in the substance of the lung.—*N. Y. Pathological Society. Med. Record.*

